# Reproduction numbers for epidemic models with households and other social structures. I. Definition and calculation of *R*_0_

**DOI:** 10.1016/j.mbs.2011.10.009

**Published:** 2012-01

**Authors:** Lorenzo Pellis, Frank Ball, Pieter Trapman

**Affiliations:** a MRC Centre for Outbreak Analysis and Modelling, Department of Infectious Disease Epidemiology, Imperial College London, United Kingdom; b School of Mathematical Sciences, University of Nottingham, United Kingdom; c Department of Mathematics, Stockholm University, Sweden

**Keywords:** SIR epidemic, Households, Basic reproduction number, *R*_0_, Workplaces, Branching processes

## Abstract

The basic reproduction number *R*_0_ is one of the most important quantities in epidemiology. However, for epidemic models with explicit social structure involving small mixing units such as households, its definition is not straightforward and a wealth of other threshold parameters has appeared in the literature. In this paper, we use branching processes to define *R*_0_, we apply this definition to models with households or other more complex social structures and we provide methods for calculating it.

## Highlights

► We consider SIR epidemic models with small mixing units. ► We provide a general definition of *R*_0_ in terms of branching processes. ► We apply it to models with households or other more complex social structures. ► We provide methods for calculating it.

## 1 Introduction

The basic reproduction number *R*_0_ is a ubiquitous concept in epidemiology and its success arguably stems from its clear biological interpretation as well as from its important properties. Its definition is straightforward in randomly mixing homogeneous models, but not obvious for more complex models. The main purpose here is to define and show how to compute *R*_0_ for models with various types of social structures characterised by the presence of small mixing groups, in which quick local depletion of susceptibles may occur. The reasons why this task is not trivial are discussed in Section 2.

Throughout the paper we assume a finite population of given size, with no births, deaths or migration. Individuals may be susceptible (*S*), infectious (*I*) and recovered/removed (*R*).

In the epidemic modelling literature, models in which the population is partitioned into various types of groups and mixes at different rates within groups and between groups are often referred to as *models with multiple levels of mixing* [11]. If there is no hierarchical structure given by nested groups, the models are usually referred to as *models with two levels of mixing* [10,11]. Because the groups are not necessarily large, the use of stochastic models represents the most natural framework.

The so-called *households models* are the simplest possible type of models with two levels of mixing, where there is only one group type that partitions the entire population. The group could represent any type of closed and strongly mixing environment one is interested in modelling explicitly. However, the facts that most individuals have a household and that data about epidemics of directly transmissible infections appear clustered in households [18,19] suggest the choice of interpreting groups as households – hence the model name – is a natural one.

Although there have been studies where the household size was considered large [15,10], the most common assumption of these models is that households have a small size, and that the number of households grows as the population size grows. Various types of households models appeared in the literature [10,21,43,13], differing in the assumptions that govern the person-to-person infection process and the different types of mixing in the household and in the community.

A possible generalisation of the households model is obtained when, in addition to being member of a household and making global contacts, each individual also belongs to another mixing group. The two partitions could be nested into one another – e.g. households being part of a town, in turn being part of a country, in a hierarchical structure (see [49]) – but are often thought simply as overlapping (see also [37] for a comparison between hierarchical and overlapping structures). This model has been considered, for example, in [11] under the name of *overlapping groups model* and in [39] under the name of *households–workplaces model*. For convenience, in this paper we refer to the second group as a workplace, although it can represent any environment characterised by strong mixing (e.g. an actual workplace, a school, a peer group, etc.).

**Fig. 1:**
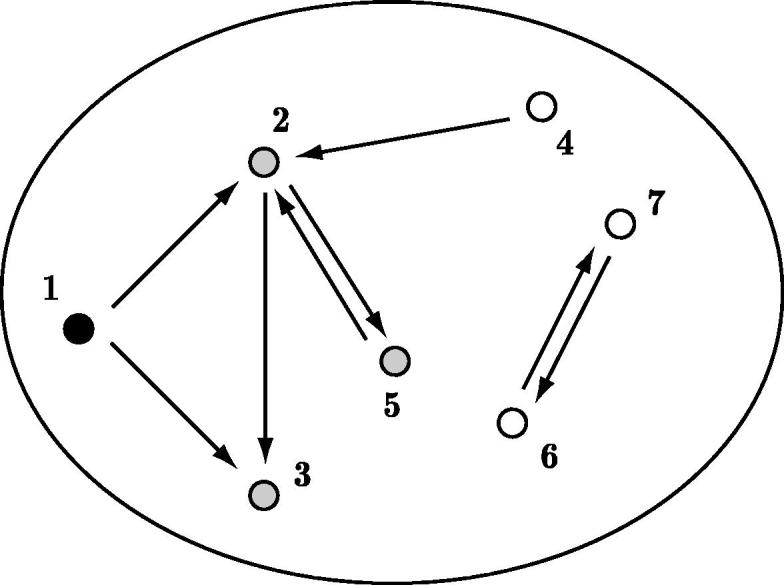
Realisation of an epidemic graph: arrows represent infectious contacts and are drawn as explained in the main text. The primary case (generation 0) is represented in black and all other cases ultimately infected are in grey. Individuals 2 and 3 are both in generation 1, individual 5 is in generation 2.

In order to define *R*_0_ for such models, we take the standard approach of approximating the early phase of the epidemic with a suitably defined branching process. To do this, individuals are placed in the generations of the branching process by constructing *the epidemic graph* (Fig. 1). Although the epidemic graph can be defined for generic epidemic models, for more clarity we introduce the standard stochastic SIR model and describe the graph construction by means of an example. Other stochastic SIR models are considered in Section 5.

In the standard SIR model, pairs of individuals contact each other independently according to Poisson processes with per-pair intensity *λ*/(*N* − 1). If an infectious individual contacts a susceptible one, then the susceptible one becomes infectious. Infectious individuals stay so for a random time, *the infectious period*. Infectious periods of different individuals are independent and identically distributed (i.i.d.) copies of a non-negative random variable I. After his or her infectious period, an individual recovers and remains immune forever.

We now create the epidemic graph as follows. The vertices of this directed graph are the individuals in the population. If, during his or her infectious period, individual *x* makes at least one contact with individual *y*, then a directed edge is drawn from *x* to *y*. For mathematical convenience we also assign “pseudo infectious periods” to those vertices that ultimately escape infection: these infectious periods are also i.i.d. copies of I. If *x* has (pseudo) infectious period $I_{x}$, then the probability that there is an edge from *x* to any other given vertex *y* is given by $1-e^{-\lambda I_{x}/(N-1)}$. Conditioned on the (pseudo) infectious period of *x*, the edges from *x* are present or absent independently of each other. By construction, the vertices that can be reached from an initial infective by a directed path correspond to the ultimately recovered individuals.

Note that, in the graph, edges appear independently, except for those with the same starting vertex, which are dependent through the infectious period (unless I is constant, in which case they are all independent of each other). Furthermore, in the epidemic graph, real time is ignored, so we keep track only of who made infectious contacts with whom and not when contacts were made or who effectively infected whom.

We define the generation of individual *x* as the minimal path length from an initial infective to *x* and we number 0 the generation of an initial infective. We note that the generation number of an individual need not coincide with the number of transmissions that led to the infection of the individual. This is the case because it is possible that a path of length *n*′ from the initial infectious individual to individual *x*, which is in generation *n* < *n*′ took less time to be formed than any of the paths of length *n* to *x*. Sometimes [33], the generation is defined as the length of the path that leads to the infection and the shortest path in the epidemic graph is then called the *rank*. We comment more on this issue in Section 5. However, for ease of exposition, unless otherwise specified we have in mind the epidemic graph construction above, where individuals are placed in generations according to their rank.

In Section 2 we recall known results about *R*_0_ for single-type and multi-type models and we discuss the difficulties in extending the concept to other social structures. In Section 3 we define *R*_0_ for the households model and present analytical results for it. The definition and results are then extended to a model with households and workplaces in Section 4. Note that throughout the paper we do not use any specific properties of models for the infectious contact process. The results discussed here are therefore very general. Specific examples of models and issues related to the definition of generation are discussed in depth in Section 5. Finally, Section 6 is devoted to concluding comments and remarks.

## 2 The concept of *R*_0_

### 2.1 Random mixing single-type populations

In this section we discuss some properties of the basic reproduction number *R*_0_ (to be defined below) for stochastic SIR epidemics in randomly mixing homogeneous populations. In SIR epidemics in a population subdivided into small households those properties can be used to define several different reproduction numbers, c.f. [22]. For a discussion on the basic reproduction number in deterministic SIR models one might consult [20].

Throughout this and the following sections, the (random) number of generation-*i* individuals in a population of size *N* is denoted by $X_{i}^{\left(N\right)}$. We also assume for convenience that there is only a single initially infectious individual, chosen uniformly at random from the population, while all other individuals are susceptible. Generalisations to other initial conditions are straightforward.

The basic reproduction number *R*_0_ for epidemics in homogeneous randomly mixing populations is defined as the expected number of individuals infected by the initially infected individuals, during their entire infectious period. That is, $R_{0}=E\left(X_{1}^{\left(N\right)}\right)$. Because the number of initially susceptible individuals is *N* − 1, *R*_0_ can be computed to be (*N* − 1)*p*, where *p* is the probability that a given initial susceptible is infected by the initial infectious individual. For example, in the standard stochastic SIR epidemic model, $R_{0}=(N-1)E(1-e^{-I\lambda /(N-1)})$. In large populations, it is unlikely that in the early stages of the epidemic infectious individuals make contacts with individuals that are no longer susceptible (see [7]) and therefore *R*_0_ is well approximated by the expected number of contacts an infected individual makes during his or her infectious period, i.e., for the standard stochastic SIR model, $R_{0}\approx \lambda E(I)$. This approximation for *R*_0_ is exact in the limit *N* → ∞.

The basic reproduction number is often easy to compute, since the number of infectious individuals in the early stages of an SIR epidemic in a large homogeneous randomly mixing population is well approximated by the number of individuals in the early stages of a branching process [7]. To be more precise, we note that for any *n*, the random vector $\left(X_{0}^{\left(N\right)}\text{,}X_{1}^{\left(N\right)}\text{,}X_{2}^{\left(N\right)}\text{,}\ldots \text{,}X_{n}^{\left(N\right)}\right)$ converges in distribution to (*X*_0_, *X*_1_, *X*_2_, …, *X*_*n*_) as *N*  → ∞, where *X*_*i*_ denotes the size of the *i*-th generation of a Galton–Watson branching process [28] for which the offspring distribution is mixed Poisson (i.e. Poisson with random parameter) with parameter distributed as λI. Here we note that Ball and Donnelly [7] proved more than only convergence in distribution of this random vector (see [24, Chapter 7] for a discussion on modes of convergence). Thus, in order to derive properties of the early stages of an SIR epidemic in a very large population, we might borrow results from the theory of branching processes [25,28]. The central observation is that *R*_0_ corresponds to the offspring mean of the Galton-Watson branching process, (*X*_0_, *X*_1_, *X*_2_, …), which approximates the epidemic process. In what follows we study this branching process and therefore we restrict ourselves to large population limits. Now, recalling that we assumed *X*_0_ = 1, application of the dominated convergence theorem, cf. the proof of [7, Theorem 3.1], yields

(2.1) $$R_{0}=E(X_{1})=\underset{N\to \infty }{\lim}E\left(X_{1}^{\left(N\right)}\right)\text{.}$$

We do not consider the boring branching processes in which every individual has exactly one child with probability 1, i.e. we assume that $P(X_{1}=1)<1$. We now discuss some of the important properties of *R*_0_.

1. The approximating branching process survives forever with strictly positive probability if and only if *R*_0_ > 1. The probability that the branching process survives corresponds to the large population limit of the probability that ultimately the number of removed individuals is of the same order as the population size *N*: the probability of *a giant outbreak*.
  As a side remark we note that *R*_0_ is not the only quantity which satisfies the threshold property for survival. This is easy to see by observing that if *f*(*x*) is a strictly increasing function with *f*(1) = 1, then *f*(*R*_0_) satisfies the same threshold condition.
2. Under mild conditions (see [28, p. 31] for exact formulations), as *k* → ∞, (*R*_0_)^−*k*^*X*_*k*_ converges almost surely (i.e. with probability 1) to a non-negative random variable W which is finite with probability 1, and equals 0 with probability equal to the extinction probability of the branching process. Note that P(W=0)=1 if *R*_0_ ⩽ 1. This relationship implies (but is not equivalent to stating) that (*X*_*k*_)^1/*k*^ converges almost surely to *R*_0_ if the branching process survives. We may interpret W as a random variable which quantifies the stochastic effects on the growth during the early generations of a branching process. In later generations the branching process has either gone extinct or the number of individuals per generation is large and the law of large numbers makes further significant stochastic effects unlikely.
  Provided the branching process survives, *X*_*k*_ ≈ *R*_0_*X*_*k*−1_ for large *k* (more precisely, *X*_*k*_/*X*_*k*−1_ converges almost surely to *R*_0_). Therefore, in this case *R*_0_ can be estimated from simulations as *X*_*k*_/*X*_*k*−1_, for large *k*. This is essentially the method used in [27].
  From the definition of the Galton–Watson branching process it follows trivially that, whether or not the branching process survives, we have $E(X_{n})={\left(R_{0}\right)}^{n}$ (and thus E(W)=1), or equivalently $R_{0}={\left(E(X_{n})\right)}^{1/n}$. In what follows we often use the asymptotic statement
  (2.2) $$R_{0}=\underset{n\to \infty }{\lim}{\left(E(X_{n})\right)}^{1/n}\text{.}$$
  We stress that the limit is only of use in later subsections, and that, in the definition above, *R*_0_ is the limit of a constant sequence. We note that for $Y_{n}=\sum_{i=0}^{n}X_{i}$, it holds that $R_{0}=\lim_{n\to \infty }{\left(E(Y_{n})\right)}^{1/n}$ as long as *R*_0_ > 1. This is used as a definition by Trapman [45,47]. In the following sections of the present paper we follow Miller [35] and use a definition of *R*_0_ in the spirit of (2.2).
3. Suppose *R*_0_ > 1. Then, if a critical fraction $p_{c}=1-\frac{1}{R_{0}}$ of the births in the branching process is suppressed, the effective reproduction number after the blocking is reduced to 1, while the branching process approximation is still valid. For epidemics, this implies that if at least a fraction *p*_*c*_ of the individuals in the population is vaccinated with a perfect vaccine, which makes vaccinated individuals no longer susceptible, then a giant outbreak occurs with probability 0. The same result holds if all individuals in the population are vaccinated with a “leaky vaccine” with efficacy at least *p*_*c*_, i.e. a vaccine that does not affect the individuals’ behaviour upon infection, but reduces the probability of a vaccinated susceptible being infected during a contact, independently between contacts, by at least a factor *p*_*c*_.

### 2.2 Random mixing multi-type populations

Similar results as for the ordinary Galton–Watson branching process hold for multi-type branching processes, in which each individual is one of a finite number *d* of types (in an epidemic context, these types could reflect, for example, age group, sexual activity or geographical location). Let *m*_*ij*_ be the expected number of type-*j* children of a type-*i* individual. Let *M* be the matrix with entries *m*_*ij*_, and assume that there exists an integer *k*, such that all entries of *M*^*k*^ are strictly positive (i.e. the branching process is *positively regular* [28, p. 89]). *R*_0_ is defined to be the largest eigenvalue of *M*, which by Perron-Frobenius theory exists and is unique (all other eigenvalues are strictly smaller in modulus), real and strictly positive. Let *v* = (*v*_1_, *v*_2_, …, *v*_*d*_) be the left eigenvector of *M* corresponding to *R*_0_, normalised so that *v*_1_ + *v*_2_ + ⋯ + *v*_*k*_ = 1; by Perron-Frobenius theory all elements of *v* are real and strictly positive. As in the single type case, the branching process survives with non-zero probability if and only if *R*_0_ > 1.

Furthermore, let ${\overset{¯}{X}}_{n}(j\text{;}i)$, be the number of type-*i* individuals in the *n*-th generation of a branching process starting with a *j* individual and ${\overset{¯}{X}}_{n}(j)=\sum_{i=1}^{d}{\overset{¯}{X}}_{n}(j\text{;}i)$. Then under mild conditions (see [28, p. 94] for exact formulations), ${\left(R_{0}\right)}^{-n}{\overset{¯}{X}}_{n}(j)$ converges almost surely to a random variable W(j) which is finite with probability 1 and strictly positive with probability equal to the survival probability of the branching process. Upon survival ${\overset{¯}{X}}_{k}(j\text{;}i)/{\overset{¯}{X}}_{k}(j)$ converges almost surely to *v*_*i*_, independently of the type *j* of the initial case.

In epidemiological terms, W(j) quantifies the stochastic effects during the early generations (i.e. when numbers are small) of an epidemic starting with a type-*j* infective. In later generations, the branching process has either gone extinct or numbers are large, stochastic effects negligible and, independently of the type *j* of the initial case, the proportions of each type in each generation are given by the components of *v* and the total number of cases grows at a per-generation multiplicative factor *R*_0_. So if $X_{n}^{\left(N\right)}(j\text{;}i)$ is the number of infected type-*i* individuals in generation *n* of an *SIR* epidemic started by one type-*j* individual in a population of total size *N* then, independently of *i* and *j* we have

(2.3) $$R_{0}=\underset{n\to \infty }{\lim}\underset{N\to \infty }{\lim}{\left(E\left(X_{n}^{\left(N\right)}(j\text{;}i)\right)\right)}^{1/n}\text{.}$$

This definition is the multi-type equivalent of (2.2). Note that the limits in (2.3) are necessary, while for single-type branching processes the limit in (2.2) was optional.

The results on vaccination in homogeneous populations still hold, as long as all types of individuals have the same vaccine response and the vaccine is administered uniformly at random (without replacement) in case of a perfect vaccine. This results in reducing *M* to (1 − *p*_*c*_)*M*, where *p*_*c*_ is the fraction of blocked infectious contacts. If the perfect vaccine is administered only to a subset of types, then a more directly useful reproduction number than *R*_0_ is the *type reproduction number* defined in [42,26].

### 2.3 Structured populations

In network-structured populations [1,36,13] and in populations subdivided into small households, in which contacts have a high frequency [10], things change dramatically. The main reason is that during the early stages of an epidemic it is likely that infectious individuals make infectious contacts with non-susceptibles: in other words, there is a local saturation effect.

In some network models branching process approximations are still useful and, because of their analytic tractability, there is a bias in the epidemic modelling literature towards these models. An important observation is that in most structured populations “the environment” of an individual who is infected during the epidemic is significantly different from the environment of an individual chosen uniformly at random. Indeed, at least one of the neighbors in the network of an infected individual is no longer susceptible, because he or she was the “infector”. In most network models in the literature (e.g. [36]), *R*_0_ should be redefined as $R_{0}=E(X_{2}|X_{1}=1)$. For this reproduction number the three main properties of *R*_0_ still hold (the second in the asymptotic sense of (2.3)), while the equality $E(X_{1})=1$ does not even define a threshold anymore.

In some other network models [13,47] and in households models, naive branching process approximations do not work at all, since the number of infectious contacts with susceptibles an infectious individual can make is dependent on the number of infectious contacts with susceptibles its “infector”, or other individuals infected earlier, made. To overcome this problem for household models, an alternative threshold parameter *R*_∗_, the household reproduction number, has been introduced and branching process approximations are now used at the level of households and not at the level of individuals (see e.g. [10]).

The household reproduction number *R*_∗_ is constructed as follows. Assume again that the epidemic process is started by one infectious individual. His or her household is referred to as the generation-0 household. As an intermediate step we consider the epidemic restricted to the generation-0 household: *the local epidemic*. The generation-1 households are the initially completely susceptible households which contain individuals infected by the infectives in the local epidemic (including the initial case) of the generation-0 household. Further generations are constructed in a similar way. Since it is unlikely that during the early stages of an epidemic a household is contacted more than once from outside, the probability of occurrence of loops not entirely included in a household in the early stages of an epidemic converges to 0 as the number of households grows to infinity. So branching process approximations may be used (e.g. see [5,8,10,13]) and *R*_∗_ is the offspring mean of the approximating branching process. Simulations reported by Ball and Lyne [9] for the standard households model, and by Ball et al. [13,14] for the network-households model, demonstrate that such households-based branching process approximations work well, even for modestly sized populations of small households.

In this paper we want, among other things, to obtain a working definition of *R*_0_ in the spirit of (2.3), that is $R_{0}≔\lim_{n\to \infty }\lim_{N\to \infty }{\left(E\left(X_{n}^{\left(N\right)}\right)\right)}^{1/n}$, where $X_{n}^{\left(N\right)}$ is defined as before. We note that *R*_0_ and *R*_∗_ are both threshold parameters for the possibility of a giant outbreak. Therefore, *R*_0_ = 1 if and only if *R*_∗_ = 1.

## 3 Households model

### 3.1 Model definition

In this section we treat in detail the definition of the basic reproduction number for stochastic models where the population is socially structured into (possibly small) households. Throughout this section we assume homogeneous mixing within each household, and in the following two subsections we assume that it is superimposed on to a background homogeneous mixing in the population at large. Section 3.4 considers the case when background infection is generated by a special type of network, the *configuration model*. Transmission in the two environments occurs via *household infections* and *global infections*, respectively. Analogously, we will use the terms *household infectious contacts*, as opposed to *global infectious contacts*.

Because the interest is on studying reproduction numbers, as highlighted in Section 2 we implicitly assume that we observe the early phase of an epidemic in a large and fully susceptible population, as all results apply to and are expressed in terms of an approximating branching process.

We conclude this section by introducing the *m* × *m* matrix

(3.1) $$A_{m}=\left[\begin{array}{ccccc} a_{0} & 1 & 0 & \cdots & 0\\ a_{1} & 0 & 1 & & 0\\ \vdots & & & \ddots & \\ a_{m-2} & 0 & 0 & & 1\\ a_{m-1} & 0 & 0 & \cdots & 0 \end{array}\right]\text{,}$$

which occurs repeatedly in the remainder of the paper.

In the next subsection, we assume that each household has the same size *n*_*H*_. We discuss variable households sizes in Section 3.3.

### 3.2 Uniform household size

#### 3.2.1 Computation of *R*_0_

Denote by *μ*_*G*_ the expected number of *global* infections an individual generates in the approximating branching process and denote by *μ*_*i*_, *i* = 0, 1, …, *n*_*H*_ − 1, the expected number of cases in generation *i* of a household epidemic started by a single initial case. By definition, *μ*_0_ = 1. We are not concerned here with how the *μ*_*i*_’s are calculated, as this depends on the specific model assumed for the person-to-person infection process. Further comments are reported in Section 5 and in Appendix A. The basic reproduction number is then given by the following theorem.

Theorem 1 *The basic reproduction number R*_*0*_ *is given by the dominant eigenvalue of the n*_*H*_ × *n*_*H*_ *matrix*

(3.2) $$A_{n_{H}}=\left[\begin{array}{ccccc} \mu_{G}\mu_{0} & 1 & 0 & \cdots & 0\\ \mu_{G}\mu_{1} & 0 & 1 & & 0\\ \vdots & & & \ddots & \\ \mu_{G}\mu_{n_{H}-2} & 0 & 0 & & 1\\ \mu_{G}\mu_{n_{H}-1} & 0 & 0 & \cdots & 0 \end{array}\right]\text{.}$$

Note that the matrix in (3.2) is of the form of *A*_*m*_ in (3.1), with *m* = *n*_*H*_ and *a*_*i*_ = *μ*_*G*_*μ*_*i*_, *i* = 0,1, …, *n*_*H*_ − 1. The proof is deferred to Section 3.2.3.

Corollary 1 *R*_*0*_ *can be computed as the only positive root of the function*

(3.3) $$g_{n_{H}}(\lambda )=1-\overset{n_{H}-1}{\underset{i=0}{\sum }}\frac{\mu_{G}\mu_{i}}{\lambda^{i+1}}\text{.}$$

Proof By direct expansion of the determinant of *A*_*m*_ − *λI*, we find that the characteristic polynomial *f*_*m*_(*λ*) of *A*_*m*_ satisfies the recursive relation

$$f_{m}(\lambda )=-\lambda f_{m-1}(\lambda )+{\left(-1\right)}^{m-1}a_{m}\text{,}\;m=2\text{,}3\text{,}\ldots$$

Therefore, by induction,

$$f_{m}(\lambda )={\left(-1\right)}^{m}\left[\lambda^{m}-\overset{m}{\underset{i=1}{\sum }}a_{i}\lambda^{m-i}\right]\text{,}\;m⩾1\text{.}$$

Defining *g*_*m*_(*λ*) = (−1)^*m*^*f*_*m*_(*λ*)/*λ*^*m*^ we obtain Corollary 1.

The existence and uniqueness of the root follows from the fact that *g*_*m*_(*λ*) is a monotonically increasing function of *λ*, for *λ* > 0, with lim_*λ*↓0_ *g*_*m*_ (*λ*) = −∞ and lim_*λ*→∞_ *g*_*m*_ (*λ*) = 1. □

#### 3.2.2 Intuitive argument on how *R*_0_ is constructed

Intuitively, the computation of *R*_0_ proceeds as follows. In the infinite population limit and during the early phase of the epidemic, each household is infected from outside only once. The primary case then starts a household epidemic, leading to other infectives.

Because of the small household size and therefore the quick depletion of susceptibles, the primary case is more likely to generate new infections in the household, compared to an individual infected later on in the household epidemic. Therefore, a *typical* infectious case should intuitively come from a suitable defined average of individuals infected during a household epidemic. Note that a simple arithmetic mean of the expected number of cases that each case generates during a household epidemic (both in the global population and within the household) leads to a quantity introduced in [22] as *R*_*HI*_. Denoting by *μ*_*L*_ the expected total number of infected individuals (excluding the initial infective) within a household, the expected number of within-household infections per infectious individual is *μ*_*L*_/(*μ*_*L*_ + 1), since only the initial infective within the household was infected from the outside, whence $R_{\mathrm{HI}}=\mu_{G}+\mu_{L}(1+\mu_{L})$. This approach does not lead to *R*_0_ (this was already noted in [22]): since each case in a household can make global infections and generate new primary cases in other households, such primary cases (with more potential to infect in the household) will occur more frequently than others during a growing epidemic. The idea behind *R*_*HI*_ was also used in [46, Section 5.1] to compute *R*_0_ (in [46] denoted by *R*_∗_). However, in that paper it was incorrectly claimed to be an exact way of computing *R*_0_.

Distinguishing infectives in types depending on their possibility to infect others is also not a new idea: for example, Becker and Dietz [16,17] distinguished infectives according to how many susceptibles were available in the household. The novel approach here is to distinguish cases in the household epidemic by the generation they belong to. In this case, only the expected number of cases in each generation needs to be used, owing to the fact that, during the early phase of the epidemic household epidemics evolve independently of what happens outside. A second novel element in the present work is the application of definitions (2.2) and (2.3) to households model.

Initially, Pellis proposed a more intuitive approach for the construction of the next generation matrix, which works as follows. Individuals are still distinguished in types according to the generations they are in. Now, each individual, independently of the generation, infects on average *μ*_*G*_ new household primary cases (i.e. new cases in generation 0); a household primary case infects on average *μ*_1_ cases in generation 1 in the household; the *μ*_1_ cases are responsible all together for the new *μ*_2_ cases in the following generation, so that each of them can be thought of as causing “on average” *μ*_2_/*μ*_1_ new cases; and so on.

This argument leads to an *n*_*H*_ × *n*_*H*_ next generation matrix $K_{n_{H}}=(k_{\mathrm{ij}})$, where *k*_*ij*_ represents the expected number of individuals in generation *j* infected by an individual in generation *i*, given by:

(3.4) $$K_{n_{H}}=\left[\begin{array}{ccccc} \mu_{G} & \mu_{1}/\mu_{0} & 0 & \cdots & 0\\ \mu_{G} & 0 & \mu_{2}/\mu_{1} & & 0\\ \vdots & \vdots & & \ddots & \\ \mu_{G} & 0 & 0 & & \mu_{n_{H}-1}/\mu_{n_{H}-2}\\ \mu_{G} & 0 & 0 & \cdots & 0 \end{array}\right]\text{.}$$

Note how this informal approach uses ratios of expected number of cases in subsequent generations, instead of formally dealing with random variables. However, note that $A_{n_{H}}$ does not have the biological meaning of a next generation matrix, as opposed to $K_{n_{H}}$.

Proposition 1 *The matrices* $A_{n_{H}}$ *and* $K_{n_{H}}$ *have the same dominant eigenvalue R*_*0*_*.*

Proof Consider the diagonal matrix *S*, with diagonal elements $\mu_{0}\text{,}\mu_{1}\text{,}\ldots \text{,}\mu_{n_{H}-1}$. Then, recalling that *μ*_0_ = 1, we have $A_{n_{H}}={\mathrm{SK}}_{n_{H}}S^{-1}$. Therefore $A_{n_{H}}$ and $K_{n_{H}}$ are similar and share the same eigenvalues, in particular the dominant one. □

#### 3.2.3 Proof of Theorem 1

Let *x*_*n*,*i*_, *n* = 0, 1, …, *i* = 0, 1, …, *n*_*H*_ − 1, be the expected number of cases infected in generation *n* in the epidemic at large, who are also cases in generation *i* in their household. To avoid ambiguities, we will refer to *x*_*n*,*i*_ as the mean number of individuals in *global generation n* and in *household generation i*. We refer to individuals with household generation *i* = 0 as *household primary cases* and to all other cases in a household as *household secondary cases*, even if they are not directly infected from the primary case.

Throughout this and the following sections, we adopt the convention that all variables suffixed by *n* are 0 if *n* < 0. Recall that we also assume that the epidemic starts with a single initial case.

Lemma 1 *The variables x*_*n,i*_*, n* *=* *0,* *1,* …*, i* *=* *0,* *1,* …*,* *n*_*H*_ − *1, satisfy the system of equations*

(3.5) $$x_{n\text{,}0}=\mu_{G}\overset{n_{H}-1}{\underset{i=0}{\sum }}x_{n-1\text{,}i}\text{,}$$

(3.6) $$x_{n\text{,}i}=\mu_{i}x_{n-i\text{,}0}\text{,}\;i=1\text{,}2\text{,}\ldots \text{,}n_{H}-1\text{,}$$

*with x*_*0,0*_ *=* *1 and x*_*0,i*_ *=* *0, i* *=* *1,* *2,* …*,* *n*_*H*_ − *1.*

Proof Using capital letters to denote random variables, our aim is to obtain the equations for $x_{n\text{,}i}=E[X_{n\text{,}i}]$.

In the approximating branching process, household primary cases occur only because of global infections from cases in the previous global generation but in all possible household generations. Therefore

(3.7) $$E[X_{n\text{,}0}]=E[E[X_{n\text{,}0}|X_{n-1\text{,}0}\text{,}X_{n-1\text{,}1}\text{,}\ldots \text{,}X_{n-1\text{,}n_{H}-1}]]\text{.}$$

To compute explicitly the right-hand side of (3.7), we first note that

$$X_{n\text{,}0}=\overset{n_{H}-1}{\underset{i=0}{\sum }}\overset{X_{n-1\text{,}i}}{\underset{k=1}{\sum }}Y_{n-1\text{,}i\text{,}k}^{G}\text{,}$$

where $Y_{n-1\text{,}i\text{,}k}^{G}$ represents the number of global infections generated by the *k*-th individual in global generation *n* and household generation *i*. In the branching process under consideration, the random variables $Y_{n-1\text{,}i\text{,}k}^{G}$ are all independent of *X*_*n*−1,*i*_ and i.i.d. according to the random variable *Y*_*G*_ describing the number of global contacts of an individual. From its previous definition, $\mu_{G}=E[Y_{G}]$. Then,

$$E[X_{n\text{,}0}|X_{n-1\text{,}0}\text{,}\ldots \text{,}X_{n-1\text{,}n_{H}-1}]=\mu_{G}\overset{n_{H}-1}{\underset{i=0}{\sum }}X_{n-1\text{,}i}\text{,}$$

and

(3.8) $$E[X_{n\text{,}0}]=\mu_{G}\overset{n_{H}-1}{\underset{i=0}{\sum }}E[X_{n-1\text{,}i}]\text{.}$$

The number of cases in household generation *i* ⩾ 1 depends only on how many household primary cases were infected *i* generations before, so we know that

(3.9) $$E[X_{n\text{,}i}]=E[E[X_{n\text{,}i}|X_{n-i\text{,}0}]]\text{.}$$

More precisely, for *i* = 1, 2, …, *n*_*H*_ − 1,

$$X_{n\text{,}i}=\overset{X_{n-i\text{,}0}}{\underset{k=1}{\sum }}Y_{n-i\text{,}i\text{,}k}^{H}\text{,}$$

where $Y_{n\text{,}i\text{,}k}^{H}$ represents the number of cases in generation *i* of a household epidemic started by the *k*-th household primary case among those in global generation *n*. Because in the branching process approximation the evolution of a household epidemic is independent of what happens outside, the random variables $Y_{n\text{,}i\text{,}k}^{H}$ are all independent of *X*_*n*,0_ and identically distributed according to the random variable $Y_{i}^{H}$ describing the number of cases in generation *i* of a household epidemic. Therefore, recalling that $\mu_{i}=E\left[Y_{i}^{H}\right]$,

$$E[X_{n\text{,}i}|X_{n-i\text{,}0}]=\mu_{i}X_{n-i\text{,}0}\text{,}$$

and, by (3.9),

(3.10) $$E[X_{n\text{,}i}]=\mu_{i}E[X_{n-i\text{,}0}]\text{.}$$

In conclusion, using Eqs. (3.8) and (3.10), (3.7) and (3.9) lead to the desired system for the expectations. □

For *n* = 0, 1, …, let $x_{n}=\sum_{i=0}^{n_{H}-1}x_{n\text{,}i}$ be the expected total number of cases in global generation *n* and let **x**^(*n*)^ be the vector of the expected total number of cases in *n*_*H*_ consecutive global generations up to $n\text{,}\;x^{\left(n\right)}=(x_{n}\text{,}x_{n-1}\text{,}\ldots \text{,}x_{n-n_{H}+1})$. Then the following holds.

Lemma 2 *The vectors* **x**^*(n)*^*, n* *=* *0,* *1,* …*, satisfy the recursive relation:*

$$x^{\left(n\right)}=x^{\left(n-1\right)}A_{n_{H}}\text{,}$$

*where* $A_{n_{H}}$ *is defined as in* (3.2) *and* **x**^*(0)*^ *=* *(1,* *0,* …*,* *0).*

We provide two proofs of this lemma, the first one is straightforward, but not easily generisable to more complex situations, while the second one is more involved but more general.

Proof 1 From the definition of *x*_*n*_, we can rewrite Eq. (3.5) as *x*_*n*,0_ = *μ*_*G*_*x*_*n*−1_ and, after substituting it in Eq. (3.6), we obtain *x*_*n*,*i*_ = *μ*_*i*_*μ*_*G*_*x*_*n*−1−*i*_. Summation over all *i* = 0, 1, …, *n*_*H*_ − 1 (including the case of *i* = 0 from Eq. (3.5)) gives *x*_*n*_ at the left-hand side and, recalling that *μ*_0_ = 1,

(3.11) $$x_{n}=\overset{n_{H}-1}{\underset{i=0}{\sum }}\mu_{G}\mu_{i}x_{n-1-i}\text{,}$$

which, if written in matrix form, is exactly the result needed. □

Proof 2 From Lemma 1, Eqs. (3.5) and (3.6) can be rewritten in terms of the expected number of household primary cases only, in the form

(3.12) $$x_{n\text{,}0}=\overset{n_{H}-1}{\underset{i=0}{\sum }}\mu_{G}\mu_{i}x_{n-i-1\text{,}0}\text{.}$$

In general, denote by $X_{i}^{\left(n\right)}=(x_{n\text{,}i}\text{,}x_{n-1\text{,}i}\text{,}\ldots \text{,}x_{m-n_{H}+1\text{,}i})\text{,}\;n=0\text{,}1\text{,}\ldots \text{,}\;i=0\text{,}1\text{,}\ldots \text{,}n_{H}-1$ the vector of the expected number of cases in household generation *i* in *n*_*H*_ subsequent global generations up to n. Then Eq. (3.12) can be rewritten in matrix form as

(3.13) $$x_{0}^{\left(n\right)}=x_{0}^{\left(n-1\right)}A_{n_{H}}\text{.}$$

From previous definitions we have that

$$x^{\left(n\right)}=\overset{n_{H}-1}{\underset{i=0}{\sum }}x_{i}^{\left(n\right)}$$

and from Eq. (3.6) we have

$$x_{i}^{\left(n\right)}=\mu_{i}x_{0}^{\left(n-i\right)}\text{.}$$

Therefore,

(3.14) $$x^{\left(n\right)}=\overset{n_{H}-1}{\underset{i=0}{\sum }}\mu_{i}x_{0}^{\left(n-i\right)}\text{.}$$

Iterating Eq. (3.13) yields $x_{0}^{\left(n\right)}=x_{0}^{\left(0\right)}A_{n_{H}}^{n}$, which on substituting into (3.14) gives

$$x^{\left(n\right)}=\overset{n_{H}-1}{\underset{i=0}{\sum }}\mu_{i}x_{0}^{\left(0\right)}A_{n_{H}}^{n-i}=\overset{n_{H}-1}{\underset{i=0}{\sum }}\mu_{i}x_{0}^{\left(0\right)}A_{n_{H}}^{n-1-i}A_{n_{H}}=x^{\left(n-1\right)}A_{n_{H}}.\;□$$

The proof of Theorem (2.2) is then concluded thanks to the following result.

Lemma 3 *Let ρ denote the largest eigenvalue of* $A_{n_{H}}$*. Then R*_*0*_ *=* *ρ.*

Proof First note that $A_{n_{H}}$ is positively regular. Therefore, by Perron-Frobenius theory, $A_{n_{H}}$ had a unique real and positive largest eigenvalue *ρ*, say. Denoting by **x**_*L*_ and **x**_*R*_, respectively, the left and right eigenvector corresponding to *ρ*, normalised such that **x**_*L*_**x**_*R*_ = 1, we have (see e.g. [29, pp. 542–551]) that

$$A_{n_{H}}=\rho P+B\text{,}$$

where *P* = **x**_*R*_**x**_*L*_, and (see [29, p. 547], though note the difference in notation)

(3.15) $$\rho^{-n}B^{n}\to 0\;\text{as}\;n\to \infty \text{.}$$

Thus, in the present notation with **1** denoting a column vector of ones (of suitable dimension),

$$x_{n}=x^{\left(n\right)}1=x^{\left(0\right)}A_{n_{H}}^{n}1\text{.}$$

Furthermore (see [29, p. 547]),

$$A_{n_{H}}=\rho^{n}P+B^{n}\text{.}$$

Hence,

$$x_{n}=\rho^{n}x^{\left(0\right)}P1+x^{\left(0\right)}B^{n}1=\rho^{n}\left(x^{\left(0\right)}P1+\frac{x^{\left(0\right)}B^{n}1}{\rho^{n}}\right)=\rho^{n}(x^{\left(0\right)}P1+b_{n})\text{,}$$

where, from (3.15), *b*_*n*_ → 0 as *n* →  ∞.

Thus,

$$x_{n}^{1/n}=\rho {\left(x^{\left(0\right)}P1+b_{n}\right)}^{1/n}\to \rho \;\text{as}\;n\to \infty$$

and therefore, from the definition of *R*_0_ at (2.2), *ρ* = *R*_0_. □

### 3.3 Variable household size

Consider now the case of households of different sizes. Let *n*_*H*_ now denote the largest size of a household and let *p*_*s*_, *s* = 1, 2, …, *n*_*H*_, be the probability that a randomly selected household has size *s*. Then, the probability that a randomly selected individual belongs to a household of size *s* is given by

$$\pi_{s}=\frac{{\mathrm{sp}}_{s}}{\sum_{l=1}^{n_{H}}{\mathrm{lp}}_{l}}\text{,}\;s=1\text{,}2\text{,}\ldots \text{,}n_{H}\text{.}$$

The distribution {*π*_*s*_} is usually referred to as the size-biased distribution (see e.g. [10]).

Let $\mu_{i}^{\left(s\right)}\text{,}\;s=1\text{,}2\text{,}\ldots \text{,}n_{H}\text{,}\;i=0\text{,}1\text{,}\ldots \text{,}s$, be the expected number of cases in generation *i* of an epidemic started by a single initial case in a household of size *s*. Then the same results obtained for the case where households are all of the same size apply if we redefine

(3.16) $$\mu_{i}=\overset{n_{H}}{\underset{s=1}{\sum }}\pi_{s}\mu_{i}^{\left(s\right)}\text{,}$$

with the convention that $\mu_{i}^{\left(s\right)}=0$ whenever *i* ⩾ *s*. Thus, *μ*_*i*_ is now the expected number of cases in generation *i* of a single-household epidemic started by infecting an individual chosen uniformly at random from the entire population. Formal calculations involve writing the equations for the expected number of cases, $x_{n\text{,}i}^{\left(s\right)}$ say, in global generation *n* and in household generation *i* of an epidemic in a household of size *s*. Using (3.16), the system simply reduces to that of Lemma 1.

### 3.4 Network of global contacts

In the network-households model of [13,14] global contacts take place through a network generated by the configuration model, rather than occurring because of homogeneous mixing. The network is defined by attaching to each individual in the population a random number *D* of half-edges drawn, independently for distinct individuals, from a specified degree distribution, $p_{k}^{D}$ = P(*D* = *k*) (*k* = 0, 1, …) say, having finite mean *μ*_*D*_. These half-edges are then paired uniformly at random to create a network of possible global contacts. (If the total number of half-edges is odd then there is a loose half-edge that is ignored.) The allocation of degrees to individuals is assumed to be independent of the partitioning of the population into households.

Observe that, except for the initial generation, primary and secondary cases in a household typically have different degree distributions. A primary case is caused by a global contact and, owing to the way the network is constructed, for *k* = 2, 3, …, it is *k* times as likely to have degree *k* than degree 1. Thus, the degree of a typical primary case is distributed according to a random variable, $\overset{∼}{D}$ say, having degree-biased distribution $P(\overset{∼}{D}=k)={\mathrm{kp}}_{k}^{D}/\mu_{D}\;(k=1\text{,}2\text{,}\ldots )$. By contrast, a typical secondary case is infected within a household and his or her degree is distributed according to *D*. Let *p*_*G*_ be the probability that a given infectious individual transmits infection to a given global neighbour. Then, in the early phase of an epidemic, the mean number of global infections made by primary and secondary infectives are ${\overset{˜}{\mu }}_{G}=p_{G}E[\overset{∼}{D}-1]$ and *μ*_*G*_ = *p*_*G*_E[*D*], respectively. (For a primary case, one of his or her edges is connected to the infector and hence is not available for disease transmission.) A simple calculation shows that, provided $p_{G}>0\text{,}\;{\overset{˜}{\mu }}_{G}=\mu_{G}$ if and only if the mean and variance of *D* are equal, as is the case when *D* has a Poisson distribution (when in fact *D* and $\overset{∼}{D}-1$ have the same distribution).

The derivation of *R*_0_ proceeds exactly as described above for the standard households model, the only difference being that now, in the matrix *A*_*m*_ of (3.1), $a_{0}={\overset{˜}{\mu }}_{G}$ (for all other *i* = 1, …, *n*_*H*_ − 1, we still have *a*_*i*_ = *μ*_*G*_*μ*_*i*_).

## 4 Households–workplaces model

### 4.1 Model definition

In this model, we assume that each individual belongs to a household and a workplace. Both places are referred to as *local* environments, and in each of them we assume homogeneous mixing. The global mixing is also assumed to be homogeneous, although some generalisations like the one used in the previous subsection can be included in the present formalism.

A possible representation of the social structure of this model is through a bipartite network (see e.g. [39]): each node can be of only one of two types, representing households and workplaces; an edge between a household and a workplace represents an individual belonging to both of those environments; edges between two households or two workplaces are not allowed.

In general it is hard, if not impossible to make useful branching process approximations even during the early stage of the epidemic in a large population, because the bipartite network connecting households and workplaces might present some short loops and parallel edges (see [11,39]). Therefore, in general it is very hard, to compute a meaningful reproduction number for this model.

As done in [11,39], however, we assume here that the probability of loops of bounded length in the bipartite network vanishes as the population size tends to infinity. In other words, loops of local contacts can occur only within the same household or workplace. A sufficient condition for this, for example, is that every individual chooses his or her workplace at random among all workplaces (each one weighted by its own size). In the infinite population limit, this assumption makes the bipartite network locally tree-like and allows a branching process to be embedded in the early stages of the epidemic.

### 4.2 Computation of *R*_0_

For ease of exposition, assume that all households have the same size *n*_*H*_ and all workplaces have the same size *n*_*W*_. In addition, define *n*_*T*_ = *n*_*H*_ + *n*_*W*_. Extensions to variable sizes are straightforward and dealt with as in Section 3.3.

As for the households model, denote by *μ*_*G*_ the expected number of global infections an individual generates during the early phase of the epidemic in a large and otherwise fully susceptible population and denote by $\mu_{i}^{H}\text{,}\;i=0\text{,}1\text{,}\ldots \text{,}n_{H}-1$, and by $\mu_{j}^{W}\text{,}\;j=0\text{,}1\text{,}\ldots \text{,}n_{W}-1$, the expected number of cases, respectively, in generation *i* of a household epidemic and generation *j* of a workplace epidemic, each started by a single initial case. By definition, $\mu_{0}^{H}=\mu_{0}^{W}=1$. As for the households model, we do not comment here on how the $\mu_{i}^{H}$s and $\mu_{j}^{W}$s are calculated (see Section 5, instead). The basic reproduction number is then given by the following theorem.

Theorem 2 *The basic reproduction number R*_*0*_ *for the model with households and workplaces is given by the dominant eigenvalue of the (n*_*T*_ − *1)* × *(n*_*T*_ − *1) matrix*

(4.1) $$A_{n_{T}}=\left[\begin{array}{ccccc} c_{0} & 1 & 0 & \cdots & 0\\ c_{1} & 0 & 1 & & 0\\ \vdots & & & \ddots & \\ c_{n_{T}-3} & 0 & 0 & & 1\\ c_{n_{T}-2} & 0 & 0 & \cdots & 0 \end{array}\right]\text{,}$$

*where, for* $k=0\text{,}1\text{,}\ldots \text{,}n_{T}-2$*,*

(4.2) ck=μG∑i+j=k0⩽i⩽nH-10⩽j⩽nW-1μiHμjW+∑i+j=k+11⩽i⩽nH-11⩽j⩽nW-1μiHμjW.

Corollary 2 *R*_*0*_ *for the households–workplaces model can be calculated as the unique positive root of the function:*

$$g_{n_{T}}(\lambda )=1-\overset{n_{T}-2}{\underset{k=0}{\sum }}\frac{c_{k}}{\lambda^{k+1}}\text{,}$$

*with the c*_*k*_ *defined in* (4.2)*.*

The proof of this corollary is exactly the same as that of Corollary 1.

### 4.3 Proof of Theorem 2

We extend the ideas developed in the simple case of the households model. We however refrain from providing the details about the computation with the random variables and work directly with the expectations. The argument involving random variables is in line with the one presented for the households model; in particular, taking expectations is straightforward, because household and workplace epidemics are independent from global infections in the branching process approximating the early phase of the epidemic and are independent of each other as there are no finite loops in the network connecting households and workplaces.

**Fig. 2:**
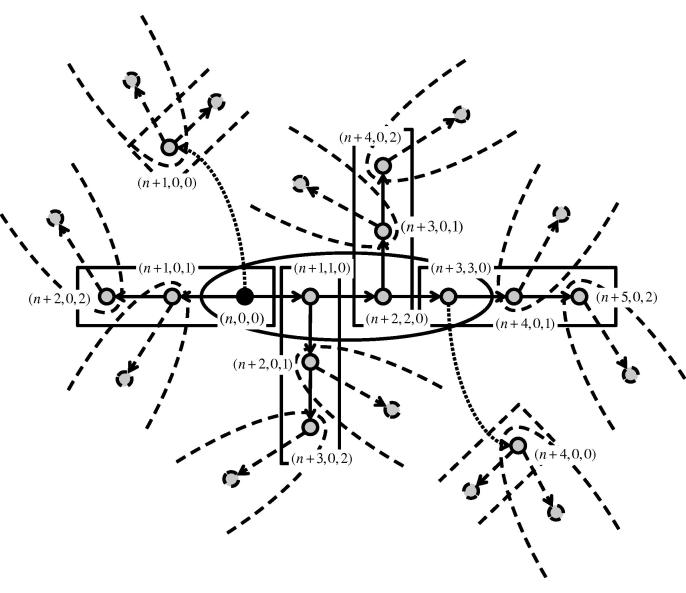
Schematic representation of the labelling convention for the households–workplaces model. Each label (*n*, *i*, *j*) indicates that the individual it refers to contributes to the count of the variable *x*_*n*,*i*,*j*_. The infection tree is rooted in a single double primary case in generation *n* (black dot). Ellipses represent households and rectangles workplaces. In this example, each individual generates (if possible) one following case in each of the two local structures. In addition, two individuals make a global contact each (curved dotted arrow), generating two new double primary cases. Lines become dashed when reaching the boundaries of the figure. Labels are added until a new household or double primary case is generated. Further household infections reset indices (*n* + *k*, *i*, *j*) to (*n* + *k*, 1, 0), for suitable *k*.

Let the system variables be of the form *x*_*n*,*i*,*j*_, where the first index refers to *global generation*, the second to the *household generation* and the third to the *workplace generation* (see Fig. 2). More specifically, in each global generation *n* ⩾ 0,

1. *x*_*n*,0,0_ represents the number of individuals that have been infected via a global infection: following [39], these will be referred to as *double primary cases*, because they can trigger an epidemic both in their household and in their workplace;
2. *x*_*n*,0,*j*_, *j* = 1, 2, …, *n*_*W*_ − 1 represents the number of cases in generation *j* of a workplace epidemic: these will be referred to either as *workplace secondary cases* or *household primary cases*, because they can trigger an epidemic in their household but not in their workplace;
3. *x*_*n*,*i*,0_, *i* = 1, 2, …, *n*_*H*_ − 1 represents the number of cases in generation *i* of a household epidemic: these will be referred to either as *household secondary cases* or *workplace primary cases*, because they can trigger an epidemic in their workplace but not in their household.

As highlighted in Fig. 2, this distinction characterises all possible individuals in the branching process, because loops may be ignored in the social structure. Therefore, we do not consider the variables *x*_*n*,*i*,*j*_ with both *i* ≠ 0 and *j* ≠ 0. As in Section 3, we adopt the convention that all variables suffixed with *n* are 0 for *n* < 0 and we assume that the epidemic starts with a single initial case.

Observe that arguments similar to the ones used to prove Lemma 1 lead to the system of equations

(4.3) $$x_{n\text{,}0\text{,}0}=\mu_{G}\left(x_{n-1\text{,}0\text{,}0}+\overset{n_{H}-1}{\underset{i=1}{\sum }}x_{n-1\text{,}i\text{,}0}+\overset{n_{W}-1}{\underset{j=1}{\sum }}x_{n-1\text{,}0\text{,}j}\right)\text{,}$$

(4.4) $$x_{n\text{,}0\text{,}j}=\mu_{j}^{W}\left(x_{n-j\text{,}0\text{,}0}+\overset{n_{H}-1}{\underset{i=1}{\sum }}x_{n-j\text{,}i\text{,}0}\right)\text{,}\;j=1\text{,}2\text{,}\ldots \text{,}n_{W}-1\text{,}$$

(4.5) $$x_{n\text{,}i\text{,}0}=\mu_{i}^{H}\left(x_{n-i\text{,}0\text{,}0}+\overset{n_{W}-1}{\underset{j=1}{\sum }}x_{n-i\text{,}0\text{,}j}\right)\text{,}\;i=1\text{,}2\text{,}\ldots \text{,}n_{H}-1\text{,}$$

with *x*_0,0,0_ = 1 and *x*_0,*i*,*j*_ = 0, *i* = 1, 2, …, *n*_*H*_ − 1, *j* = 1, 2, …, *n*_*W*_ − 1.

Let now $x_{n\text{,}0}=\sum_{j=1}^{n_{W}-1}x_{n\text{,}0\text{,}j}$ be the expected total number of household primary cases in global generation *n* and let *y*_*n*,0_ = *x*_*n*,0,0_ + *x*_*n*,0_ be the number of all those that can start an epidemic in a household. Furthermore, denote by $y_{0}^{\left(n\right)}\text{,}\;n⩾0$, the vector of the total expected number of cases that can start a household epidemic in *n*_*T*_ consecutive global generations up to $n\text{,}\;y_{0}^{\left(n\right)}=(y_{n\text{,}0}\text{,}y_{n-1\text{,}0}\text{,}\ldots \text{,}y_{n-n_{T}+1\text{,}0})$. Then the following holds.

Lemma 4 *The vectors* $y_{0}^{\left(n\right)}\text{,}\;n=0\text{,}1\text{,}\ldots$ *satisfy the recursive relation:*

$$y_{0}^{\left(n\right)}=y_{0}^{\left(n-1\right)}A_{n_{T}}\text{,}$$

*where* $A_{n_{T}}$ *is defined as in* (4.1) and (4.2)*, and* $y_{0}^{\left(n\right)}=(1\text{,}0\text{,}\ldots \text{,}0)$*.*

Proof Substituting Eq. (4.5) into Eqs. (4.3) and (4.4), we can express the system only in terms of double primary or household primary cases:

$$\begin{array}{cc} & x_{n\text{,}0\text{,}0}=\mu_{G}\left(x_{n-1\text{,}0\text{,}0}+\overset{n_{H}-1}{\underset{i=1}{\sum }}\mu_{i}^{H}\left(x_{n-1-i\text{,}0\text{,}0}+\overset{n_{W}-1}{\underset{k=1}{\sum }}x_{n-1-i\text{,}0\text{,}k}\right)+\overset{n_{W}-1}{\underset{j=1}{\sum }}x_{n-1\text{,}0\text{,}j}\right)\text{,}\\ & x_{n\text{,}0\text{,}j}=\mu_{j}^{W}\left(x_{n-j\text{,}0\text{,}0}+\overset{n_{H}-1}{\underset{i=1}{\sum }}\mu_{i}^{H}\left(x_{n-j-i\text{,}0\text{,}0}+\overset{n_{W}-1}{\underset{k=1}{\sum }}x_{n-j-i\text{,}0\text{,}k}\right)\right)\text{.} \end{array}$$

Using the definition of *x*_*n*,0_ and summing the second equation over index *j*, we obtain

(4.6) $$x_{n\text{,}0\text{,}0}=\mu_{G}\left(x_{n-1\text{,}0\text{,}0}+\overset{n_{H}-1}{\underset{i=1}{\sum }}\mu_{i}^{H}(x_{n-1-i\text{,}0\text{,}0}+x_{n-1-i\text{,}0})+x_{n-1\text{,}0}\right)\text{,}$$

(4.7) $$x_{n\text{,}0}=\overset{n_{W}-1}{\underset{j=1}{\sum }}\mu_{j}^{W}\left(x_{n-j\text{,}0\text{,}0}+\overset{n_{H}-1}{\underset{i=1}{\sum }}\mu_{i}^{H}(x_{n-j-i\text{,}0\text{,}0}+x_{n-j-i\text{,}0})\right)\text{.}$$

Recalling that $\mu_{0}^{H}=1$ and using the definition of *y*_*n*,0_ , the system can be rewritten as

$$\begin{array}{cc} & x_{n\text{,}0\text{,}0}=\mu_{G}\overset{n_{H}-1}{\underset{i=0}{\sum }}\mu_{i}^{H}y_{n-1-i\text{,}0}\text{,}\\ & x_{n\text{,}0}=\overset{n_{W}-1}{\underset{j=0}{\sum }}\mu_{j}^{W}x_{n-j\text{,}0\text{,}0}+\overset{n_{W}-1}{\underset{j=1}{\sum }}\mu_{j}^{W}\overset{n_{H}-1}{\underset{i=1}{\sum }}\mu_{i}^{H}y_{n-j-i\text{,}0}\text{.} \end{array}$$

Adding the two equations together (*y*_*n*,0_ = *x*_*n*,0,0_ + *x*_*n*,0_), substituting *x*_*n*−*j*,0,0_ from the first equation into the second one and recalling that $\mu_{0}^{W}=1$, finally leads to a closed equation in the *y*s:

(4.8) yn,0=μG∑0⩽i⩽nH-10⩽j⩽nW-1μiHμjWyn-1-i-j,0+∑1⩽i⩽nH-11⩽j⩽nW-1μiHμjWyn-i-j,0,

which, when expressed in matrix form, is exactly the statement required. □

Let $x_{n}=\sum_{i\text{,}j:\mathrm{ij}=0}x_{n\text{,}i\text{,}j}$ be the expected total number of cases in global generation *n* and let **x**^(*n*)^, *n* = 0, 1, … the vector of the expected total number of cases in *n*_*T*_ consecutive global generations up to *n*. Then we have the following.

Lemma 5 *The vectors* **x**^*(n)*^*, n* *=* *0,* *1,* … *satisfy the recursive relation:*

$$x^{\left(n\right)}=x^{\left(n-1\right)}A_{n_{T}}\text{,}$$

*where* $A_{n_{T}}$*is defined in Eqs.* (4.1) and (4.2)*, and* **x**^*(0)*^ *=*  *(1,* *0,* …*,* *0).*

Proof Similarly to *x*_*n*0_ in Eq. (3.12), $y_{n\text{,}0}$ is the key variable for which we are able to obtain a closed equation, namely Eq. (4.8). The aim is now to write *x*_*n*_ in terms of the *y*_*n*,0_s.

Because $x_{n}=y_{n\text{,}0}+\sum_{i=1}^{n_{H}-1}x_{n\text{,}i\text{,}0}$ and, from Eq. (4.5), $x_{n\text{,}i\text{,}0}=\mu_{i}^{H}y_{n-i\text{,}0}$, Eq. (4.8) directly gives us:

xn=μG∑0⩽i⩽nH-10⩽j⩽nW-1μiHμjWyn-1-i-j,0+∑1⩽i⩽nH-10⩽j⩽nW-1μiHμjWyn-i-j,0.

In matrix form, we have

x(n)=μG∑0⩽i⩽nH-10⩽j⩽nW-1μiHμjWy0(n-1-i-j)+∑1⩽i⩽nH-10⩽j⩽nW-1μiHμjWy0(n-i-j)

and, since $y_{0}^{\left(n\right)}=y_{0}^{\left(0\right)}A_{n_{T}}^{n}$,

x(n)=μG∑0⩽i⩽nH-10⩽j⩽nW-1μiHμjWy0(0)AnTn-1-i-j+∑1⩽i⩽nH-10⩽j⩽nW-1μiHμjWy0(0)AnTn-i-j=μG∑0⩽i⩽nH-10⩽j⩽nW-1μiHμjWy0(0)AnTn-2-i-j+∑1⩽i⩽nH-10⩽j⩽nW-1μiHμjWy0(0)AnTn-1-i-jAnT=x(n-1)AnT.□

With the same considerations for the proof of Theorem 1, the irreducibility of $A_{n_{T}}$ implies that for *n* → ∞, the normalised components of **x**^(*n*)^ converge to those of the left eigenvector corresponding to the dominant eigenvalue *ρ* of $A_{n_{T}}$. The latter is therefore the asymptotic multiplicative factor for each element of **x**^(*n*)^, in particular for the first element *x*_*n*_, and hence *ρ* = *R*_0_. This concludes the proof of Theorem 2.

## 5 Number of cases in each generation

The fundamental ingredient at the base of all results in this paper is the expected number *μ*_*i*_ of cases in each generation of an epidemic. Because these quantities have a biological meaning that is independent of the assumed model for the person-to-person contact process, the results obtained are very general. However, in order to compute them as a function of more basic parameters, such a model needs to be specified. We have already discussed the standard SIR model in the Introduction. In this section, we provide an overview of other possible model choices and discuss the related issues.

### 5.1 Discrete-generation models

Arguably, the simplest possible framework is given by the Reed–Frost model. In this model, an individual is assumed to experience a latent period (infected but not infectious) of a fixed length, usually taken as the time-unit in the model, after which all the transmission is concentrated in a single point in time. Each susceptible independently escapes infection from each infective with fixed probability *q*. Therefore, it is straightforward to construct the epidemic graph described in Section 2.

The Reed–Frost model can be extended to the so-called *randomised Reed–Frost* model, where the one-to-one escaping probabilities from different infectives are independent realisations of a random variable *Q* having a specified distribution [3,41]. A further generalisation of the randomised Reed–Frost model is the so-called *collective Reed–Frost* model [41,32] and a very general approach has also been considered by Scalia-Tomba [44].

Since the epidemic spreads in discrete steps, even if we do not know who is responsible for the infection of whom in the following generation, infectives are naturally partitioned into generations. Appendix A reports some techniques for the efficient computation of the expected number of cases in each generation for these models.

### 5.2 Continuous-time models

Although the above models can sometimes provide a useful approximation to realistic infections with a long latent period (e.g. measles), their main drawback lies in their unrealistic temporal dynamics. Alternative models for the person-to-person contact process that have appeared in the literature are usually based on the assumption that infections can occur in continuous time. In a stochastic framework, virtually all continuous-time models assume that infectives make infectious contacts at the points of a Poisson process with specified rate (for details about Poisson processes, see e.g. [24, p. 246–250] and followings). The difference between the models lies in the different assumptions about the infection rate.

The standard SIR model met in the Introduction (see also [2,4]) is arguably the most studied stochastic continuous-time model. Another possible model that has appeared in the literature (already in [30]) is sometimes referred to as the *time-since-infection model*. It has been mostly studied in a deterministic framework [20], although recently it has been treated stochastically in some households models [21,22,40]. In this model it is assumed that, after infection, an individual makes infectious contacts with other randomly selected individuals at the points of a Poisson process with time-inhomogeneous infection rate described by a function *β*(*τ*), identical for each infective, where *τ* represents the time elapsed since the infection of the individual. The function *β*(*τ*) is assumed to be non-negative, with *β*(*τ*) = 0 for *τ* < 0 and, for biological reasons, $R_{0}=\int_{0}^{+\infty }\beta (\tau )d\tau$ is assumed to be finite.

Further extensions, where the infectivity profile *β*(*τ*) is random or is different in different environments (e.g. within- versus between-households), are not commonly treated in the literature (but see, for example, [22] or [40]).

### 5.3 Overlapping generations

**Fig. 3:**
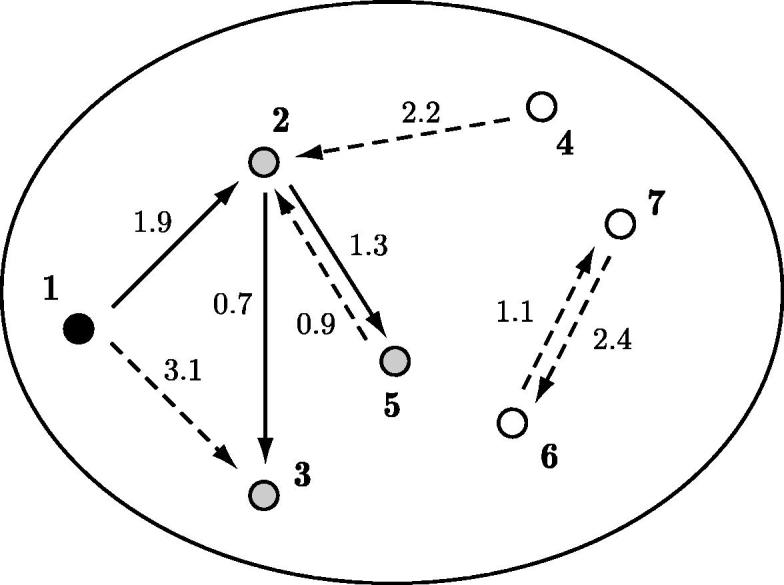
The same epidemic graph as in Fig. 1, but with dashed arrows representing infectious contacts that fail to lead to an infection, while solid arrows represent real transmission events, which depend on the time of occurrence. The weight attached to an arrow from *i* to *j* represents the time elapsing from the infection of *i* to the first infectious contact from *i* to *j*. In this example, if the initial case is infected at time *t* = 0, individual 2 infects individual 3 at time *t* = 1.9 + 0.7 = 2.6, i.e. before the infectious contact from individual 1 to individual 3. Thus, individual 3 has rank 1 (can be reached from individual 1 through a single infectious contact in the epidemic graph), despite having been infected through a chain of two transmission events (i.e. being in real-time generation 2 in the weighted graph).

All the models described previously are characterised by the fact that the infectious behaviour of individuals can be described *a priori*, i.e. even before the epidemic starts, with the proviso that it is simply ignored for those individuals that escape infection. Therefore, the epidemic graph can be constructed as shown in the Introduction. However, during the process of construction of the epidemic graph, all information about times of infection is lost and for this reason it is not possible to reconstruct the full epidemic process from the epidemic graph. An important consequence is that the attribution of individuals to different generations according to the epidemic graph can in general be different from counting the number of transmissions in the real-time process (see Fig. 3).

The distinction between the real-time process and the epidemic graph was first made by Ludwig [33], who introduced the concept of *rank* to indicate the generation to which infectives are artificially attributed in the epidemic graph described in the Introduction. The fundamental insight provided by Ludwig is that, as long as the epidemic graph can be constructed *a priori*, even if generation and rank of individuals do not necessarily coincide, the epidemic final size distribution and the final size distribution as computed from the epidemic graph are identical (see [38] for the conditions under which the graph can be constructed *a priori*). In other words, in all the above models, the epidemic graph leads to the correct epidemic final size, but in continuous-time models it can happen, for example, that a tertiary case (generation 2) in the real-time epidemic is subsequently contacted by a primary case (generation 0 and rank 0), therefore appearing from the graph to be of rank 1 (see Fig. 3). This phenomenon is often referred to as the problem of *overlapping generations*. See [38] for further details.

We do not distinguish between rank and generation throughout the paper because this distinction concerns only the preliminary stage involving the person-to-person infectious contact process used to compute the *μ*_*i*_s. Once the *μ*_*i*_s are determined, we refer to them as mean generation sizes, in the standard branching process terminology.

Note that the attribution to generations using individuals’ ranks as for the epidemic graph shown in the Introduction leads to a basic reproduction number *R*_0_ which depends only on whether or not infectious contacts occur, and not on when they occur. The alternative choice of attributing generations counting the number of real-time transmissions, instead, leads to an *R*_0_ that is in general dependent on the detailed assumptions governing the dynamics of the infectious contacts. A possible way of constructing it is to create another graph, where each link between two individuals has a weight (see Fig. 3), corresponding to the time interval between the infection of the first of them and the first infectious contact towards the other (In the statistical physics literature this model is known as *first-passage percolation* [23, p. 369]). The generation of an individual is then given by the number of links in the path with the total minimum sum of the weights of all links that connect an initial infective to the individual considered (more details are discussed in [12]).

Given that, in simple random mixing models, *R*_0_ is independent of the details concerning times on contacts, it is tempting to privilege the former construction over the latter. However, it must be acknowledged that all simple random mixing models assume no local saturation of susceptibles: therefore, in a large population and in the early phase of the epidemic, both graph constructions above contain short loops with negligible probability and thus attribute the same individuals to the same generations. Therefore, the distinction between the two constructions is of importance only in models with small mixing groups.

There is no reason why the dependence only on the integrated infectivity over the infectious period, as opposed to the full infectivity profile, should be a *defining* property of *R*_0_ in simple models, instead of being merely a *consequence* of the model assumptions. Therefore, when small mixing groups are introduced, both the epidemic graph and the weighted graph constructions are equally valid, despite the resulting *R*_0_s being different. Indeed, more generally, different methods of attributing individuals to generations lead to different reproduction numbers (which, in general, would not even have the same interpretation as *R*_0_). This and related issues, such as which choice of generation model is most relevant for prediction/control analysis, are discussed in our sequel paper [12]. In particular, we show in that paper that all methods of attributing individuals to generations lead to the same predictions concerning whether or not a large outbreak is possible; i.e. all these reproduction numbers are simultaneously larger than, smaller than or equal to 1. In the present context, this implies that if *R*_0_ > 1 when constructed from the epidemic graph, then *R*_0_ > 1 also when constructed from the weigthed graph, and similarly for *R*_0_ < 1 and *R*_0_ = 1. Here we suggest (and have in mind) a “details-independent” definition of *R*_0_ based on the rank construction proposed by Ludwig [33], but this choice is only dictated by mathematical convenience and personal taste.

## 6 Conclusions

In this paper we have proposed a working definition of *R*_0_ for models with households or households and workplaces. For these types of models, many threshold parameters with various biological meanings and purposes have been defined in the literature. Comparisons between them and *R*_0_ is matter of ongoing work [12]. However, as far as the basic reproduction number is concerned, there is somewhat of a general debate on how to extend its definition from the simplest case of homogeneously mixing model to models where the presence of small mixing groups is responsible for local saturation of susceptibles early on in the epidemic. We hope that the present work provides some clarification in this direction. We discuss here the possible reasons for such difficulties and recapitulate the argument behind the definition and construction of *R*_0_ suggested here.

In the simple single-type homogeneously mixing model, *R*_0_ is loosely defined as the expected number of cases infected by a single case, throughout his or her entire infectious period, in a large and fully susceptible population. The large and fully susceptible population assumption is required because it allows the epidemic dynamics, intrinsically non-linear, to be linearised during the early phase of the epidemic. Therefore, the initial spread can be approximated by a branching process (a linear process, because the offspring distribution remains the same at every generation). The approximation works well until the epidemic starts experiencing global saturation of susceptibles, when the non-linearity of the infection process cannot be ignored any longer.

Seen from another perspective, in order to define *R*_0_ and have it (an average value) “driving” the epidemic process, a time-scale separation is required between the average duration of the infectious period and the duration of the entire epidemic. Although from a mathematical point of view the linearisation process (or the time-scale separation) can only be made rigorous in the limit of an infinite population, in any finite populations, it is in the very first generation that the linear dynamics represent the best approximation of the true dynamics. For this reason, the intuitive definition of *R*_0_ is very often phrased in terms of an introduction of a newly infected individual in an otherwise susceptible population. Although such a view is appropriate in the case of the single-type homogeneously mixing model, we argue that sticking to it too literally in the definition of *R*_0_ may have been the cause for part of the difficulties in extending it to more complex models.

As already acknowledged in Section 2.3, in a households model, it is not possible in general to define *R*_0_ in terms of the infectious behaviour of the very first individual in the population *and* require at the same time that it be a threshold parameter, because the initial case is also a household primary case and has more potential to infect than any other secondary case.

We deemed the threshold property of *R*_0_ more relevant than its description in terms of the very first case in the population because, as highlighted above, the latter is only a way of phrasing the need to look at the epidemic when global saturation of susceptibles is still negligible.

Although this idea seems somewhat natural and it is indeed clearly well accepted when dealing, for example, with multitype models with random mixing or network models, for some reasons it seems not to be equally straightforward with households models. We therefore explicitly draw the parallel between these models in what follows.

Extending the definition of *R*_0_ to multitype models requires adjusting the loose definition above by referring to the average number of cases generated by a “typical” infectious case. This opens the question of what a “typical” case really is. The correct approach has been suggested by Diekmann and Heesterbeek [20]: a *typical* case is a “mixture” of cases of different types, in proportions given by the eigenvector relative to the dominant eigenvalue of the next generation matrix. The behavior of the first individual is irrelevant in the long term, as generation after generation the proportions of cases of each type tend to stabilise of the components of the eigenvector. In order to “see” *R*_0_, it is therefore necessary to wait for that time window that is sufficiently late for the initial condition to have been “forgotten”, but sufficiently early to be still able to ignore the global saturation of susceptibles.

Nevertheless, it is common perspective to think that the idea of looking at the behavior of the very first case may still apply, if one accepts (somewhat arguably) to start the epidemic with a typical case in the sense above.

With network models for which the number of cases grows exponentially in the early phase (e.g. in the configuration model), a typical case is charaterised by the fact that one of his neighbours cannot be infected any longer, because he or she was the source of the infection. Therefore one needs to wait at least until generation 1, before being able to find a typical case.

In a households model, one needs to wait until enough cases have been infected that a typical case can arise. Because individuals differ in the potential to infect others according to when they are infected in the household epidemic, one needs to wait at least until an entire household has been infected. Therefore the matrix $A_{n_{H}}$ of Theorem 1 has dimension equal to the maximum size of a household. The idea developed in the paper is the correct way of taking into account that, while a household epidemic is raging, their members infect other households, so cases of different type appear in different proportions and are affected both by the within- and the between-household infectivity. Note that now the linearisation occurs at the level of households, thus requiring that the number of households is large, i.e. that there is a time-scale separation between the duration of a household epidemic and the duration of the entire epidemic.

The same idea applies to the model with households and workplaces, where now the smallest complete sub-epidemic lasts as long as a full household epidemic followed by the latest possible workplace epidemic triggered by an infective in that household. Therefore, the matrix $A_{n_{T}}$ of Theorem 2 has dimension equal to the sum of the maximum household and workplace sizes minus 1 (to allow for an overlap between household and workplace epidemics).

This argument suggests that the method introduced in this paper can be extended to any model for which it is possible to identify a sub-unit of the social structure such that a time-scale separation between a sub-epidemic raging through it and the full epidemic can be assumed. (An example of a sub-unit that exists in principle, but for which no time-scale separation can in general be assumed, is the *clump*, as introduced in [11].)

In particular, a rapid investigation of the sub-epidemic in such a sub-unit, in the style of Fig. 2, provides also a tool to write the coefficients in the matrix *A*_*m*_ directly, without the need to derive the equations manually as in Sections 3.2.3 and 4.3, thus making this approach easily generalisable to many other social structures.

Given the generality of this method once the *μ*_*i*_s have been determined, one might also notice that choosing other ways to attribute cases to generations, different from the one used here to obtain *R*_0_, can lead to other known reproduction numbers. We extensively develop this approach in [12], but it is already straightforward to see that placing in generation 0 all cases in the household epidemic (i.e. setting *μ*_0_ = 1 + *μ*_*L*_ and *μ*_*i*_ = 0 for *i* = 1, 2, *n*_*H*_ − 1) leads to the dominant eigenvalue of $A_{n_{H}}$ being equal to *R*_∗_ (see Section 2.3, or [10]).

Such generalisability is indeed backed up by the observation that Corollaries 1 and 2 are nothing else than definitions of *R*_0_ as implicit solutions of discrete-time versions of the Lotka-Euler equation (see [20, p. 103], [48] or [40]) derived from a suitably defined (Crump-Mode-Jagers) branching process (see [28], Sections 5 and 6). This elegant alternative approach is investigated further in [12].

The theory developed here can be also extended further in different directions, for example to multitype epidemic models with the same social structure considered here, or with more complex structures. Extensions to infections that do not lead to permanent immunity after recovery – e.g. households SIS models (see e.g. [6]) – are also possible, although care needs to be taken when dealing with the fact that the number of generations in a household is unbounded and the method requires the use of infinite matrices.

Finally, future research could also focus on the more general problem of comparing the behaviour of the types of models considered here and other structurally different ones. Such a comparison is often non-trivial, because different models can have different numbers of parameters with different biological meanings and it is often not obvious which epidemiological quantities is worth keeping fixed in order to make the comparison fair. Given the ubiquitous presence of *R*_0_ in the field of infectious disease epidemiology, we deem it to be one of the best available candidates for this purpose, but the lack of clear definitions and analytical tools for *R*_0_ for models involving small mixing units, have hindered their comparison with other models. We believe that the present work provides an invaluable tool in this direction.

## Appendix A Computation of the mean number of cases in each generation

Here we present methods for the computation of the expected number of cases in each generation of an epidemic in a (small) population of size *n*. We denote the probability of *m* susceptibles out of *s* escaping direct infection from *a* infectives by *P*_*a*_(*m*, *s*) (*a* = 1, 2, …, *s* = 1, 2, …, *m* = 0, 1, …, *s*). We also denote by *i* = *s* − *m* the number of infectives in the following generation.

For the case of the simple Reed–Frost model, the probabilities are binomial:

(A.1) $$P_{a}(m\text{,}s)=\left(\begin{array}{c} s\\ m \end{array}\right){\left(q^{a}\right)}^{m}{\left(1-q^{a}\right)}^{s-m}\text{.}$$

The particular case of a standard SIR model with individual-to-individual infectious rate *λ* and constant duration *ι* of the infectious period corresponds, in a generation perspective, to a simple Reed–Frost model with *q* = e^−*λι*^.

For the randomised Reed–Frost model, the probability of a susceptible escaping infection from a given infective is a random variable *Q* and, conditional on the value of *Q*, the probabilities are still binomial of the same form of (A.1), but the unconditional probabilities

(A.2) $$P_{a}(m\text{,}s)=\left(\begin{array}{c} s\\ m \end{array}\right)E[{\left(Q^{a}\right)}^{m}{\left(1-Q^{a}\right)}^{s-m}]$$

are not easy to write explicitly. Therefore, it is convenient to directly use the method developed by Picard and Lefèvre [41] for the more general *collective* Reed–Frost model.

Assuming there is no variation in susceptibility (the collective Reed–Frost model can be extended also to this case, though; see [31]), we define the probability *q*(*k*) that a set of *k* susceptibles escapes infection from a single infective, and then obtain, for fixed *a* and *s*, the probabilities *P*_*a*_(*m*, *s*) (*m* = 1, 2, …, *s*) by solving the triangular system (see [41], Equation after (2.6)):

(A.3) $$\overset{s}{\underset{m=k}{\sum }}\frac{m!}{k!}P_{a}(m\text{,}s)=\frac{s!}{k!}q{\left(k\right)}^{a}\text{,}\;k=1\text{,}2\text{,}\ldots \text{,}s\text{.}$$

The equation $P_{a}(0\text{,}s)=1-\sum_{m=1}^{s}P_{a}(m\text{,}s)$ then gives the probability for *m* = 0. Alternatively, from Eq. (9) of Martin-Löf [34], one can derive explicitly

$$P_{a}(m\text{,}s)=\left(\begin{array}{c} s\\ m \end{array}\right)\overset{s}{\underset{k=m}{\sum }}{\left(-1\right)}^{k-m}\left(\begin{array}{c} s-m\\ k-m \end{array}\right)q{\left(k\right)}^{a}\text{,}\;m=0\text{,}1\text{,}\ldots \text{,}s\text{.}$$

For the standard SIR model, $q(k)=E[Q^{k}]$, and in the particular case of exponentially distributed duration I with recovery rate *ν*, *q*(*k*) = *ν*/(*ν* + *kλ*).

The expected number of cases in each generation can now be obtained as follows. Consider an epidemic with *a* initial infectives and *s* initial susceptibles. Denote by *Y*_*a*,*s*,*k*_ the number of cases in generation *k* of this epidemic and let $\mu_{a\text{,}s\text{,}k}=E[Y_{a\text{,}s\text{,}k}]$ be its expectation. Then, conditioning on the first generation, for *a* = 1, 2, …, *s* = 1, 2, … and *k* = 1, 2, …, *s*, we obtain the recursive relation

$$\mu_{a\text{,}s\text{,}k}=E[Y_{a\text{,}s\text{,}k}]=E[E[Y_{a\text{,}s\text{,}k}|Y_{a\text{,}s\text{,}1}]]=\overset{s-k+1}{\underset{i=1}{\sum }}E[Y_{a\text{,}s\text{,}k}|Y_{a\text{,}s\text{,}1}]P\{Y_{a\text{,}s\text{,}1}=i\}=\overset{s-k+1}{\underset{i=1}{\sum }}P_{a}(s-i\text{,}s)\mu_{i\text{,}s-i\text{,}k-1}\text{,}$$

with *μ*_*a*,*s*,0_ = *a* and *μ*_*a*,0,*k*_ = 0, for *a* = 1, 2, …, *s* = 1, 2, … and *k* = 1, 2, …,*s*.

Another possible method for calculating the expected number of cases in each generation involves constructing a matrix $P_{T}=(p_{h\text{,}k}^{T})$ that describes the transition probabilities from each state *h* = (*s*, *a*) to each state *k* = (*m*, *i*), and then obtain the probabilities of each chain by matrix multiplication. If the states (*s*, *a*) are ordered for increasing *s* and, for each *s*, for increasing *a*, *P*_*I*_ takes a very convenient triangular form. Furthermore, note that full operations of matrix multiplication can be avoided because the vector *v*_*k*_, *k* = 1, …, *n* − 1 of being in each possible state in generation *k* can be computed recursively as *v*_*k*_ = *P*_*T*_*v*_*k*−1_.
